# Classifying Ingestive Behavior of Dairy Cows via Automatic Sound Recognition

**DOI:** 10.3390/s21155231

**Published:** 2021-08-02

**Authors:** Guoming Li, Yijie Xiong, Qian Du, Zhengxiang Shi, Richard S. Gates

**Affiliations:** 1 Department of Agricultural and Biosystems Engineering, Iowa State University, Ames, IA 50011, USA; 2 Department of Animal Science, University of Nebraska-Lincoln, Lincoln, NE 68588, USA; yijie.xiong@unl.edu; 3 Department of Biological Systems Engineering, University of Nebraska-Lincoln, Lincoln, NE 68588, USA; 4 Department of Electrical and Computer Engineering, Mississippi State University, Starkville, MS 39762, USA; du@ece.msstate.edu; 5 Department of Agricultural Structure and Bioenvironmental Engineering, College of Water Resources and Civil Engineering, China Agricultural University, Beijing 100083, China; shizhx@cau.edu.cn; 6 Egg Industry Center, Departments of Agricultural and Biosystems Engineering, and Animal Science, Iowa State University, Ames, IA 50011, USA; rsgates@iastate.edu

**Keywords:** audio, dairy cow, deep learning, mastication, jaw movement, forage management, precision livestock management

## Abstract

Determining ingestive behaviors of dairy cows is critical to evaluate their productivity and health status. The objectives of this research were to (1) develop the relationship between forage species/heights and sound characteristics of three different ingestive behaviors (bites, chews, and chew-bites); (2) comparatively evaluate three deep learning models and optimization strategies for classifying the three behaviors; and (3) examine the ability of deep learning modeling for classifying the three ingestive behaviors under various forage characteristics. The results show that the amplitude and duration of the bite, chew, and chew-bite sounds were mostly larger for tall forages (tall fescue and alfalfa) compared to their counterparts. The long short-term memory network using a filtered dataset with balanced duration and imbalanced audio files offered better performance than its counterparts. The best classification performance was over 0.93, and the best and poorest performance difference was 0.4–0.5 under different forage species and heights. In conclusion, the deep learning technique could classify the dairy cow ingestive behaviors but was unable to differentiate between them under some forage characteristics using acoustic signals. Thus, while the developed tool is useful to support precision dairy cow management, it requires further improvement.

## 1. Introduction

Modern dairy farms continue to grow in herd size and technology adoption for maintaining or improving the production and labor efficiencies needed to feed the growing human population [1]. The U.S. is the largest dairy producer in the world with 9.39 million milking cows on farms, producing 101.25 million metric tons of milk in 2020 [2]. Despite only accounting for 6.3% of the total number of dairy farms, dairy farms containing over 500 cows have a 65.9% market share of the U.S. total milking cow inventory [3]. In these intensive production systems, forage-fed dairy production comprises over 80% [4], in which forage is a source of food and nutrients for dairy cows. Thus, sufficient provision of forage is critical to meet cow daily nutrient requirements and sustain desired milk production goals. On a short-time scale, forage intake of cows has been associated with a sequence of three jaw movements or ingestive behaviors [5], namely bites, chews, and chew-bites. A biting behavior is defined as the apprehension and severance of forage, while a chewing behavior includes the crushing, grinding, and processing of ingested grass inside the mouth [6]. A chewing-biting behavior results from the overlapping of chewing and biting events in the same jaw movement, in which the forage in the mouth is chewed, and simultaneously, a new mouthful of forage is severed [7]. The frequency and characteristics of the ingestive behaviors can change in correspondence with individual animals and surrounding environments. Therefore, efficiently and precisely monitoring and assessing the ingestive behaviors of dairy cows may provide useful insights into resource management, nutrition supply, animal health, welfare, and production.

The efficiencies and accuracies of ingestive behavior monitoring can be influenced by measurement methods. Early strategies for monitoring ingestive behavior relied on observation by technicians [8], to obtain precise individual information on a few animals but is costly and impractical for monitoring large herds [9]. Imaging methods combined with image processing algorithms or deep learning techniques may provide contactless and non-invasive measures and potentially automate the detection process [1]. However, high-quality images/videos are prerequisites for this, and appropriately recording the whole jaw movement process without occlusion within herds could be problematic. Wearable sensors including pressure sensors, accelerometers/pendulums, jaw switches, and electromyography have been examined to detect jaw movements [7]. Most of the wearable sensors focus on recognizing long-term activities (grazing or ruminating) or overall jaw movements yet have difficulties differentiating bites, chews, and chew-bites. Andriamandroso et al. [7] compared and summarized three types of sensors to classify jaw movements. They demonstrated that the accuracy of detecting jaw movement was 0.91–0.95 for noseband pressure sensors, 0.94–0.95 for microphones, and 0.65–0.90 for accelerometers, but only microphone data supported differentiating bites, chews, and chew-bites and with a relatively low accuracy of 0.61–0.95. The great potential of acoustic signals for ingestive behavior monitoring lies in the fact that ingestive sounds can be clearly transmitted from bones, skull cavities and soft tissues to recording devices typically attached to animal foreheads. Additionally, the wearable sound collecting devices do not influence cattle’s natural behaviors once the animals have acclimated to the devices [6].

Several automatic recognition systems based on acoustic signals have been developed to detect and classify the three ingestive behaviors of dairy cows. Acoustic signals of dairy cows suggest that the ingestive behavior characteristics (e.g., intake rate, bite mass, bite rate, etc.) are associated with forage species (e.g., alfalfa and tall fescue) and grass heights (e.g., tall and short) [5,10], which provide critical suggestions on precision forage management for dairy cows. However, relationships between forage species/heights and key features of acoustic signals of the three ingestive behaviors remain unclear and should be explored to supplement knowledge for precision dairy management, such as grass utilization estimation, grass preference evaluation, and other forage management. In previous work, conventional machine learning models or knowledge-based algorithms have been examined [6,11,12,13,14], including random forest, support vector machine, multi-layer perceptron, decision logic algorithm, and hidden Markov model. Despite great performance, these methods require thorough designs of feature extractors to obtain appropriate features (e.g., duration, amplitude, spectrum, and power). Alternatively, deep learning techniques (e.g., convolutional neural network, CNN; and recurrent neural network, RNN) developed from conventional machine learning are representation-learning methods and can automatically discover features from raw data without extensive engineering knowledge on feature extraction [15]. Thus, these techniques may have the potential to automatically classify the ingestive behaviors of dairy cows based on acoustic signals, but the performance of various architectures requires further investigation.

The objectives of this research were to (1) examine the effects of forage species and forage height on key acoustic characteristics of bites, chews, and chew-bites for dairy cows; (2) evaluate deep learning models and optimization strategies for automatic sound recognition of these three ingestive behaviors; and (3) examine the efficacy of a deep learning model for classifying the three behaviors for various forage characteristics. 

## 2. Materials and Methods

### 2.1. Dataset Description

**Figure 1:**
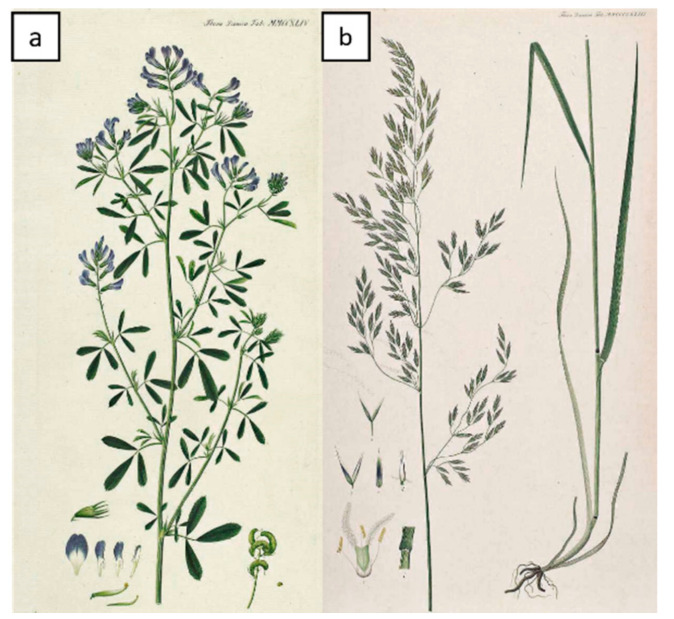
Generic illustration of the two forages described in this manuscript—(**a**) Alfalfa (*Meicago sativa)* and (**b**) *tall fescue (Lolium arundinaceum*, Schreb.) Sources: plantillustration.org.

A publicly available dataset of dairy cows was used in this research [16]. The dataset contains data collected using three microphones (Nady 151 VR, Nady Systems, Oakland, CA, USA) attached to the foreheads of three 4- to 6-year-old lactating Holstein cows weighing 608 ± 24.9 kg; and ingesting sounds (i.e., chews, bites, and chew-bites) from four types of microswards were continuously recorded for five days. The microswards consisted of sets of 4-L plastic pots with either alfalfa (*Meicago sativa*) or tall fescue (*Lolium arundinaceum*, Schreb.) with two heights, tall (24.5 ± 3.8 cm) or short (11.6 ± 1.9 cm). An illustration of the two forages used in the dataset is provided in Figure 1. The acoustic signals were saved as WAV files and labeled for observed ingestion behavior by the technicians from the same research team with a labeling agreement of over 99%. A total of 52 labeled WAV files totaling 54 min 24 s were in the dataset, and the files were processed and segmented based on ingestive behaviors, forage species, and forage height, resulting in 3038 segments with a duration of 1647.37 s being used for model evaluation and experiments (Table 1).

**Figure 2:**
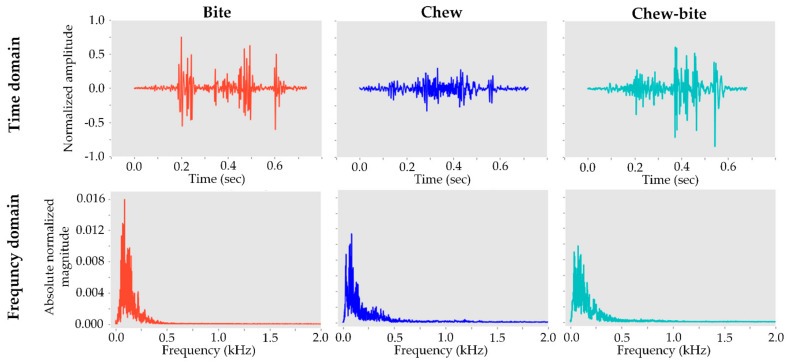
Sample illustrations of acoustic signals in time (**top**) and frequency (**bottom**) domains for the ingestive sounds. Higher absolute normalized values indicate higher power of the acoustic signal.

Sample illustrations of acoustic signals in frequency and time domains are presented in Figure 2 for the three ingestive behaviors. The three behaviors showed apparent differences in temporal and spatial signal patterns, and the acoustic signals of the behaviors were mostly in low frequencies (<1 kHz) [14]. Additionally, based on the observation reports from the technicians, each ingestive behavior event commonly lasted for less than 1 s [16]. The recorded acoustic signals are in 16 bit sample depth format taken at a 22.05 kHz sampling rate. There were $2^{16}=65,536$ possible values for each recorded signal ranging from −32,768 to 32,767. For Figure 2, amplitude values in the time domain were normalized using Equation (1), and the magnitudes in the frequency domain were normalized using Equation (3).

(1) $$Normalized\;amplitude=Recorded\;amplitude/2^{16}$$

(2) $$FFT\;value=FFT.RFFT\left(Normalized\;amplitude\right)$$

(3) $$ANM=ABS\left[{\left(FFT\;value\right)}_{i}\right]/\overset{m}{\underset{i}{\sum }}ABS{\left(FFT\;value\right)}_{i}$$

where *FFT* is Fast Fourier Transform; *RFFT* is Real Fast Fourier Transform; the function *FFT.RFFT* is to transform the discrete time domain signals into discrete frequency domain components; *ANM* is absolute normalized magnitude; *ABS* is absolutization operation; ${\left(FFT\;value\right)}_{i}$ is the $i^{\mathrm{th}}$ *FFT* value normalized; and *m* is total number of FFT values converted and its length is dynamically determined by an audio segment input. Normalized amplitude and ANM range from 0 to 1. All operations in the above equations were vectorized to improve calculation efficiency. 

### 2.2. Statstical Analysis for Evaluating Effects of Forage on Acoustic Features

Statistical analyses were conducted to investigate the relationships between forage species and heights and acoustic features of the ingestive behaviors. The data used for statistical analysis were all in the time domain. The normalized amplitude and duration of the acoustic signals were extracted for each of the 3038 segments. The acoustic feature labels provided with the dataset were processed by the research team [16]. A larger normalized amplitude indicates that louder sounds were produced around cow mouths; and duration is the length of a segment, with a longer duration indicating dairy cows spent more time ingesting forage [16]. The effects of forage species, forage heights, and their interaction on the amplitude and duration of the segments corresponding to the three ingestive behaviors were analyzed with ANOVA using PROC MIXED in Statistical Analysis Software (version 9.3, SAS Institute Inc., Cary, NC, USA). Mean values were compared using Fisher’s least significant difference with PDMIX800 [17], and a significant difference was considered at *p* ≤ 0.05. The statistical model is the same for the three behaviors of bites, chews, and chew-bites, which can be expressed as

(4) $$Y_{ijk}=\mu +\alpha_{i}+\beta_{j}+{\left(\alpha \beta \right)}_{ij}+\varepsilon_{ijk}$$

where $Y_{ijk}$ is the parameter examined (i.e., amplitude and duration); μ is the least square mean of the parameter; $\alpha_{i}$ is the forage species, i=alfalfa, tall fescue; $\beta_{j}$ is the forage height, j=tall, short; ${\left(\alpha \beta \right)}_{ij}$ is the interaction effect of forage species and height; and $\varepsilon_{ijk}$ is the random error. 

### 2.3. Overall Deep Learning Algorithm Workflow 

**Figure 3:**
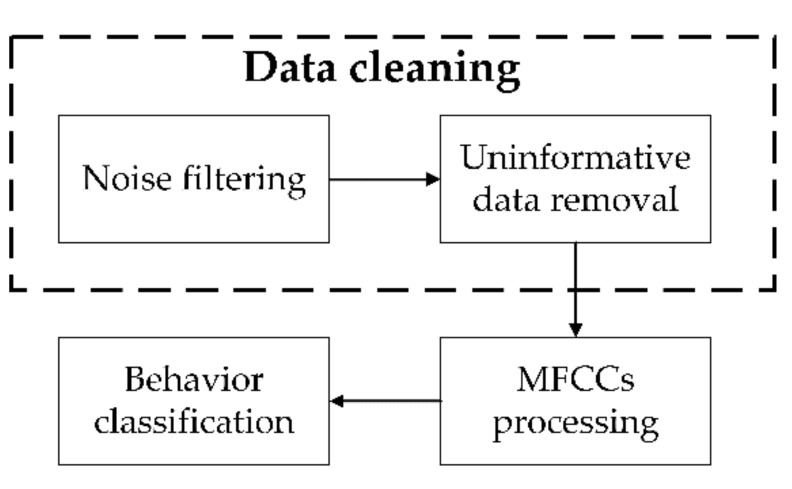
Overall workflow of the algorithms. MFCC is Mel-frequency cepstral coefficient.

As shown in Figure 3, the overall workflow consisted of four steps. The first step was to filter background noises (i.e., beeping sounds) from the input acoustic data in order to reduce interference for classification. The second step was to remove the low power signals, which can be considered as uninformative data. This may help to constrain model attention to learn important features and improve inference efficiency and accuracy. The first two steps were to clean data based on physical characteristics in the dataset. However, unwanted signals and low-power signals still existed after the initial two steps of data cleaning. The third step was to convert cleaned data into Mel-frequency cepstral coefficient (MFCC) features which are used to highlight high-power data and transform the original acoustic spectrogram into the human perception level [18]. The first three steps all involved filtering, but only the first step was named “filtering” to differentiate the data cleaning procedures. The final step was to classify the ingestive behaviors using the processed data and deep learning models. Details of the four steps are elaborated in Section 2.4, Section 2.5 and Section 2.6. The processing was conducted in a local machine with the processor of Intel(R) Core (TM) i9-10900KF CPU @ 3.7GHz, installed memory (RAM) of 128 GB, graphics processing unit (GPU) of NVIDIA GeForce RTX 3080, and Python-based computing environments. 

### 2.4. Data Cleaning 

#### 2.4.1. Noise Filtering

**Figure 4:**
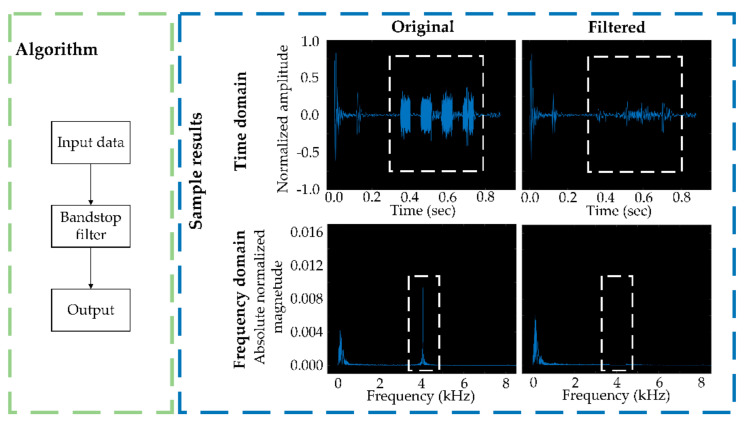
Illustration of the algorithm and sample results for noise removal. White dotted rectangles indicate period and frequency for device-related beeping before and after filtering.

A routine beeping sound associated with the recording device was produced and recorded along with the cow sounds and was randomly dispersed in the dataset, and was removed to reduce interference with detection results (Figure 4). This beeping center-frequency ranged from 3.6 to 4.5 kHz and could be effectively distinguished from cow ingesting sounds. A bandstop filter with a stopband frequency range of 3.6–4.5 kHz was used to exclude beeping sounds and maintain cow sounds. 

#### 2.4.2. Uninformative Data Removal

**Figure 5:**
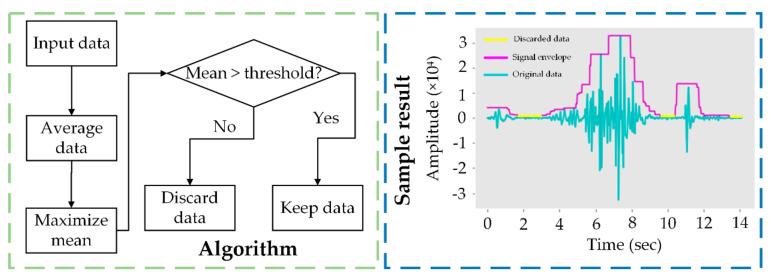
Illustration of the algorithm for uninformative data removal (**left**) and a sample result with a signal envelope after uninformative data removal (**right)**. The amplitude values are in 16 bits and unitless.

The noise-filtered dataset was then further processed to remove uninformative data. To maximize the removal efficiency, data in 16 bits without normalization of time domain were used (Figure 5). The input data were vectorized and averaged for every 1100 acoustic samples. Several means were obtained at various steps of averaging but only the maxima within groups were retained. If the maximal mean was smaller than a threshold, the input data with the corresponding index were set FALSE and discarded once converted to frequency domain. Based on the preliminary verification, a threshold value of 100 was used as it can cover most uninformative data while reducing signal loss. Nothing needed to be supplemented for the discarded data in time domain. Because in later step, the MFCC for behavior classification is in frequency domain, and a lack of a specific part in time domain did not influence the workflow for the behavior classification.

### 2.5. Mel-Frequency Cepstral Coefficients Processing

**Figure 6:**
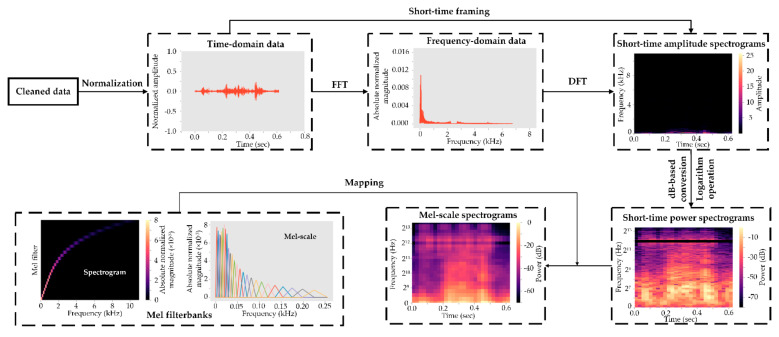
Overall workflow for the Mel-frequency cepstral coefficient processing. FFT is Fast Fourier transform, and DFT is Discrete Fourier Transform. Multiple mel-scale spectrograms were produced based on the short-time framing on time-domain signals.

The MFCC processing workflow involved several steps as summarized in Figure 6. The short-time Fourier transform was first conducted to generate a short-time amplitude spectrogram. Acoustic signals were assumed to be constant within short-time scales, and a rolling window with a length of 23 ms (512 samples) and step of 10 ms (220 samples) ran through each time-domain signal to the end of an audio file. Each windowed frame was transformed to a frequency-domain signal using a Fast Fourier transform. To disconnect adjacent overlapping frames, the Discrete Fourier Transform was operated for each frame, and a short-time amplitude spectrogram was produced accordingly. The amplitude spectrogram was converted to dB-based mel-spectrogram for human-interpretable ranges, and values in the *y*-axis were log-transformed for enhanced visualization, resulting in the short-time power spectrogram. A total of 26 Mel filterbanks were generated to retain more signals of lower frequency (which fits observed cow acoustic characteristics). The Mel filterbanks were mapped to the power spectrogram for building Mel-scale spectrograms, which were fed into deep learning models for behavior classification.

### 2.6. Architectures of Deep Learning Models 

**Figure 7:**
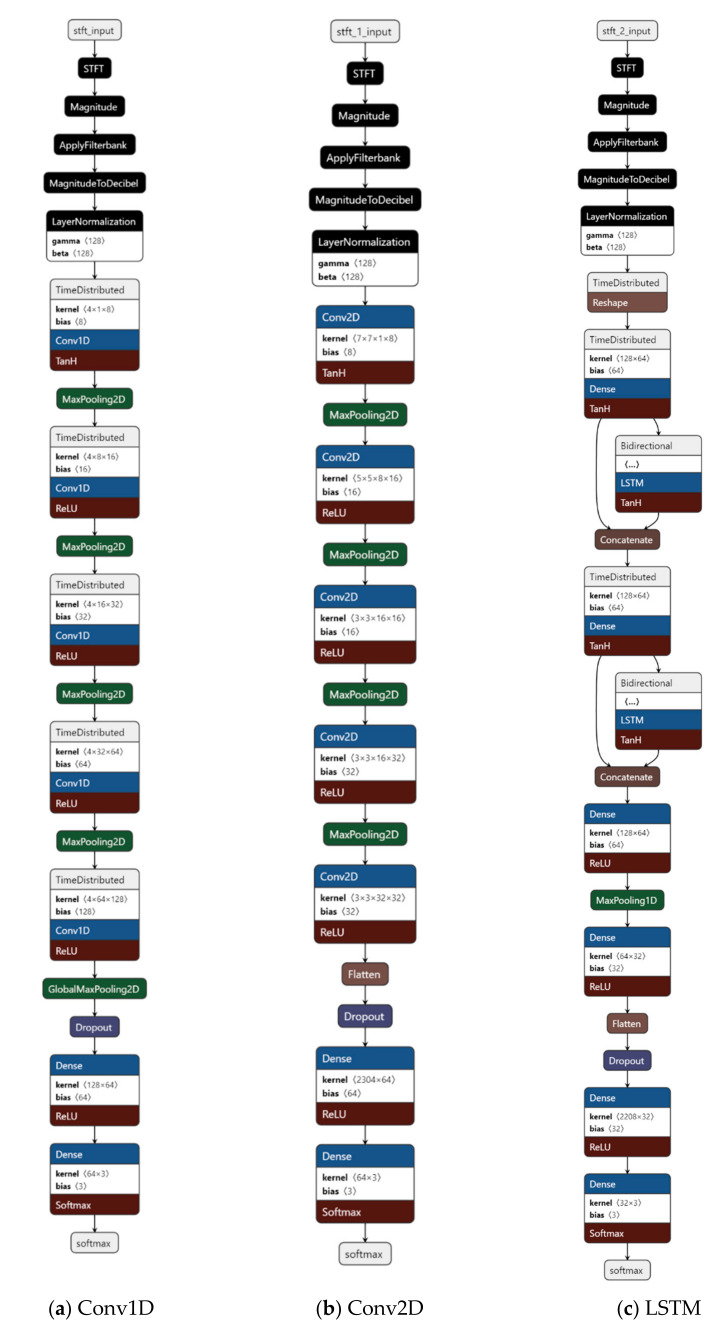
Architectures of the three proposed deep learning models for ingestive behavior classification. Conv1D represents one-dimensional convolutional neural network; Conv2D is two-dimensional convolutional neural network; LSTM represents long short-term memory network; and STFT is short-time Fourier transform.

Three deep learning models, one-dimensional CNN (Conv1D), two-dimensional CNN (Conv2D), and long short-term memory network (LSTM), were evaluated to classify acoustic data (Figure 7) [19].

The core component of Conv1D was the time-distributed layer. When a Mel-scale spectrogram was input into the model, the signal frequency at each time slot of the spectrogram was convolved in the time-distributed layer to extract high-level features. The model included a sequence of a time distributed layer and then a max-pooling layer to reduce acoustic signal dimensionality. Finally, dense and softmax layers were used to flatten two-dimensional features and connect target classes (the three ingestive behaviors). The model size and number of parameters were 706 kB and 348,770, respectively.

The Conv2D took the whole Mel-scale spectrogram as input and extracted major features through two-dimensional convolution. The structure was similar to that of Conv1D, in which a convolution layer followed by a max-pooling layer was repeatedly used to reduce dimensionality. The flatten, dense, and softmax layers were also used at the end of the network. The model size and number of parameters were 2056 kB and 431,290, respectively.

The third network was mainly constructed with two serial LSTM units. In each LSTM unit, a time distributed layer was to extract spectrogram features across time and skipped the next-layer connection; then a bidirectional RNN layer was to obtain features both in forward states (i.e., next frame of spectrogram) and backward states (i.e., previous frame of spectrogram); and finally, features from the time distributed layer and bidirectional RNN layer were concatenated to reinforce key components. After the two adjacent LSTM units, several dense layers and one flatten/softmax layer were built. The model size and number of parameters were 1862 kB and 392,050, respectively. 

### 2.7. Optimization for Classifying the Ingestive Behaviors 

The classification performance of the ingestive behaviors was optimized by comparing the three models (Conv1D, Conv2D, and LSTM), two filtering strategies (original vs. filtered), and two data organization methods (imbalanced vs. balanced). The three models were trained with a dropout rate of 0.1, activation functions of relu/tanh, and training epochs of 30. The dataset was randomized into training, validation, and testing sets with a ratio of 0.7:0.1:0.2. Training and validation accuracy curves were calculated across epochs to judge whether models were underfitted/overfitted in real time, and the hold-out dataset was used for the final testing. The filtered dataset resulted from the noise filtering methods mentioned in Section 2.4.1. Although duration of audio files in Table 1 was similar for the three ingestive behaviors, the number of audio files was different (i.e., imbalanced), which may lead to biased inference for the class (i.e., chew) with a large proportion of data. The dataset was reshuffled and randomized, and the number of audio files was equalized to 521 for the three ingestive behaviors, resulting in a balanced dataset. After data reshuffling, the duration of the audio files was 547.040 s for bites, 184.460 s for chews, and 328.180 s for chew-bites. 

### 2.8. Evaluation of Classification Performance under Various Forage Characteristics 

After optimization, the optimal model, filtering strategy, and data organization method were further used to evaluate the classification performance for ingestive behaviors under various forage characteristics. Two forage species (alfalfa vs. tall fescue) and two forage heights (short vs. tall) were compared for the three ingestive behaviors. The model was trained based on forage species and heights, using the similar training hyperparameter configurations described in Section 2.7. 

### 2.9. Evaluation Metrics

Three evaluation metrics were calculated using Equations (5)–(7), and higher values of the metrics indicate better performance.

(5) $$Precision=\frac{True\;positive}{True\;positive+False\;positive}$$

(6) $$Recall=\frac{True\;positive}{True\;positive+False\;negative}$$

(7) $$F1\;score=2\times \frac{Precision\times Recall}{Precision+Recall}$$

where *true positive* is the number of cases in which models match manual labeling; *false positive* is the number of cases in which models wrongly predict behavior presence; *false negative* is the number of cases in which models wrongly predict behavior absence. 

Based on the *true positive*, *false positive*, and *false negative*, confusion matrixes were calculated to indicate class-level performance. Diagonal values in a matrix indicate correct classification rates, and higher values suggest better class-level performance, whereas off-diagonal entries are related to misclassification. 

Processing time was reported by Python after all audio files were processed, and processing speed was normalized by dividing the processing time by the total duration of audio files tested. 

## 3. Results 

### 3.1. Ingestive Sound Characteristics under Various Forage Characteristics 

Table 2 shows the mean characteristics (i.e., amplitude and duration) and results of the statistical analysis for the bite, chew, and chew-bite behaviors with two forage species and heights. Overall, the amplitude and duration of the acoustic data were 0.323–0.488 and 0.152–0.212 s for bites, 0.084–0.117 and 0.073–0.148 s for chews, and 0.343–0.549 and 0.230–0.301 s for chew-bites, respectively. Except for the amplitude of the chewing sound, the amplitude and duration of the three ingestive sounds were larger for tall fescue than for alfalfa (*p* < 0.01). Between the two forage heights compared, tall forage resulted in larger sound amplitude and duration (excluding amplitudes of bites and chew-bites) (*p* < 0.01). Interaction effects of the forage species and heights on ingestive sounds were observed for all parameters examined. Alfalfa, a tender forage, had a lower value for all behaviors and for both amplitude and duration. The values were also greater for tall alfalfa than for short alfalfa, but both tended to be less than for the tall fescue regardless of its height. These results demonstrate that the three ingestive behaviors for the two forage species and two forage heights can be differentiated by acoustic sound characteristics, namely the amplitude and duration for specific ingestive behavior. The characteristics of the bite, chew, and chew-bite sounds of dairy cows were distinct under various forage characteristics, which could be indications of precision forage management, therefore, model ability to classify the sounds of ingestive behaviors under different forage characteristics should be further evaluated. 

### 3.2. Performance for Classifying the Ingestive Behaviors 

**Figure 8:**
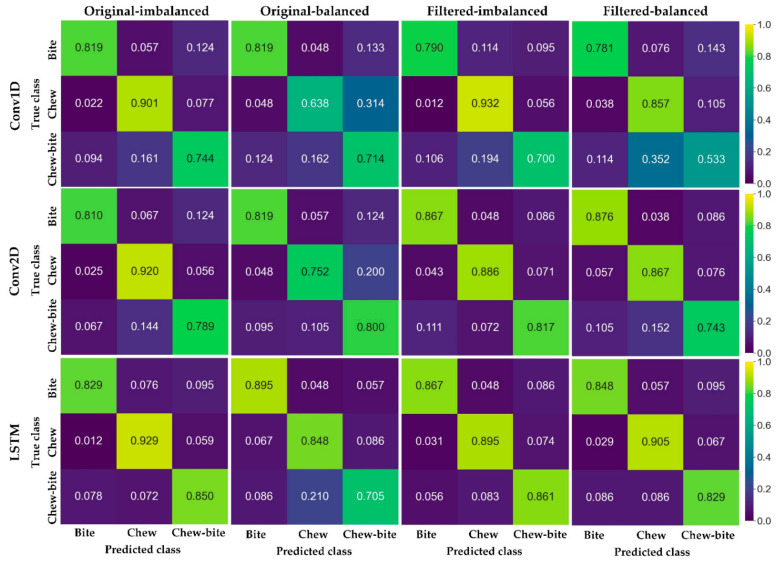
Confusion matrixes of the three deep learning models for classifying the ingestive behaviors in various datasets. Conv1D represents one-dimensional convolutional neural network; Conv2D represents two-dimensional convolutional neural network; and LSTM represents long short-term memory network. “Original” indicates the original dataset without any filtering; “Filtered” indicates the dataset was filtered with the bandstop filter to remove background beeping sounds; “imbalanced” indicates the dataset with unequal audio file sizes for the three ingestive behaviors; and “balanced” indicates the dataset with equal audio file sizes for the three ingestive behaviors.

The performance for classifying the ingestive behaviors using three deep learning models is summarized in Table 3 and Figure 8. The *precision*, *recall*, and *F*1 *score* ranged from 0.615–0.941, 0.533–0.932, and 0.599–0.932 for classifying the ingestive behaviors. The *precision*, *recall*, and *F*1 *score* of the LSTM were averagely 0.08 higher than those of Conv1D, and 0.02 higher than those of Conv2D. Overall, the classification performance for the original dataset was similar to that of the filtered dataset with a <0.01 difference on average, while the average performance for the imbalanced dataset was 0.07 higher than that for the balanced dataset. Based on the confusion matrixes (Figure 8), chewing behavior was more accurately classified than biting and chewing-biting behaviors. The Conv1D, Conv2D, and LSTM spent 70.048–74.419, 59.408–75.120, 85.366–88.035 ms for processing 1-s acoustic data. The LSTM with the filtered and imbalanced dataset was selected for further development because of better performance and comparably faster processing speed for classifying the three ingestive behaviors.

### 3.3. Performance for Classifying the Ingestive Behaviors under Various Forage Conditions

**Figure 9:**
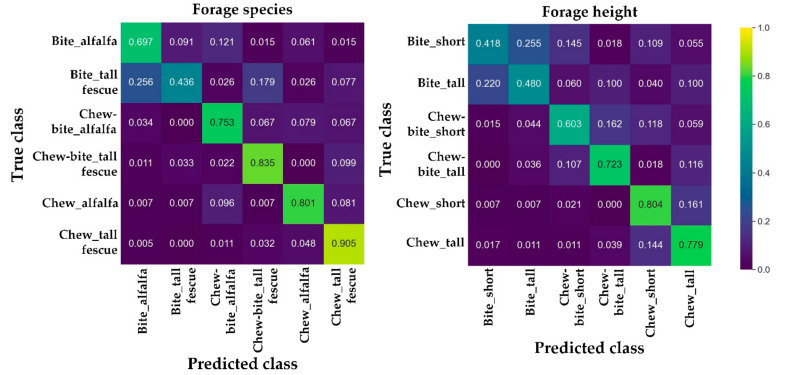
Confusion matrixes for classifying the ingestive behaviors under various forage characteristics. The model performance was investigated with the long short-term memory model and the filtered and imbalanced dataset.

The classification performance to identify particular ingestive behaviors associated with key forage characteristics was further investigated with the LSTM and filtered-imbalanced dataset (Table 4 and Figure 9). The overall *precision*, *recall*, and *F*1 *score* were 0.1–0.2 lower than those in Section 3.2. On average, the LSTM had similar classification performance for the two forage species (0.758 for alfalfa and 0.738 for tall fescue) and heights (0.620 for short and 0.620 for tall). By contrast, the overall classification performance of the ingestive behaviors for the forage species was approximately 0.1 higher than for the two forage heights. As for forage species (Figure 9), classifying biting behavior under tall fescue had the lowest accuracy (0.436) while classifying chewing under tall fescue had the highest accuracy (0.905). As for forage heights, the highest (0.804) and lowest (0.418) accuracies were observed when classifying chewing and biting for short forage.

## 4. Discussion

### 4.1. Effects of Forage on Ingestive Sound Characteristics 

Dairy cows can generate different sounds when ingesting grass materials (e.g., different grass/hay species, multiple heights, etc.) based on this research and previous investigations [5]. Understanding such information, especially the relationship between the forage characteristics and ingestive behaviors, is important to provide good cattle grazing strategies with appropriate welfare status. The ingestive sounds can be further linked with forage intake for providing supplemental information on precision management of grass utilization and animal health status [5,20]. Variations in ingestive sound characteristics may be attributed to grass/feed bulk density [21], dry matter content [20], surface area [22], diurnal pattern of intake [10], and many other options e.g., relative sheer strength of the forage. However, due to a lack of detailed information about forage characteristics, individual information, and animal status in the open-access dataset, the actual reason for the variations remains unclear and should be researched in the future. 

### 4.2. Overall Classification Performance 

The overall positive classification performance of this study and previous literature is presented in Table 5. The performance of previous studies was mainly obtained with machine learning models or knowledge-based models. One possible reason for the slightly reduced performance of the current study was the inclusion of all performance cases in the analysis (i.e., Conv1D, Conv2D, balanced dataset, etc.). With the optimal case (LSTM with filtered and imbalanced dataset), the classification performance (0.820–0.867 for bites, 0.895–0.935 for chews, and 0.824–0.861 for chew-bites) outperformed or was comparable to previous studies. The successful classification could be attributed to obvious differences in the acoustic signals among bites, chews, and chew-bites, robust data cleaning, and appropriate design of model architectures. Current forage characteristics may have little influence on model performance improvement. Perhaps, more diverse forage characteristics should be included in future research for optimizing model performance. 

### 4.3. Deep Learning Models 

To the authors’ knowledge, this paper is the first to assess the application of deep learning models for classifying dairy cow ingestive behavior sounds. During model development, key features for effective classification were learned directly from the dataset, and exhaustive labeling and dedicated manual design were not required for feature extractors that are typically required in machine learning or knowledge-based algorithms [24], enabling scientists in other domains without extensive computer science expertise applying deep learning techniques. Deep CNNs are good at handling acoustic signals because of their efficient computation and powerful learning ability [15,25]. In this study, the Conv1D and Conv2D did not perform as well as the LSTM, due to two possible reasons. Firstly, more efficient and accurate connection schemes (e.g., residual connection [26], inception connection [27], etc.) were not applied in the CNNs. Secondly, the LSTM can learn backward and feedforward features from acoustic signals, which is critical for dealing with sequential data [28]. Besides acceptable detection accuracy, decent processing speed (85.366–88.035 ms for processing 1-s acoustic data) was also achieved by the LSTM. Although powerful computing devices (with GPU of RTX 3080) are crucial components, the extremely light weight (≤2 MB) of the network architectures was the primary factor for the fast processing speed [29]. Because current networks can balance detection accuracy and processing speed, they offer new opportunities for real-time monitoring of animal conditions [30], behaviors [31], etc. for cattle industry. 

### 4.4. Other Factors Influencing Classification Performance 

Other possible influencing factors should be considered as well for future model development of sound classification. Currently, deep learning experts are switching their attention from model-centric to data-centric to improve detection performance [32]. Data quality plays a crucial role in deep learning, where improving the model hits a bottleneck now. For instance, particularly for acoustic datasets, data challenges of noise, balance, and quantity must be addressed. Considerable data noise can downgrade model performance to varying degrees. The dataset was recorded in a controlled environment with minimal introduced noises, and therefore, there was no significant model performance improvement after noise filtering. However, such a controlled environment with minimal background noise is hard to achieve in on-farm or in-field conditions. Imbalanced datasets can result in biased inference for classes with the larger proportions [33]. However, in this study the balanced dataset with the same number of audio files but uneven durations had poorer performance than the imbalanced dataset. Thus, the length of audio files may play a more important role in classification improvement than audio file quantity when selected for balancing classes. Uneven duration of audio files also downgraded the performance for different forages. Sufficient data is necessary to explore the optimal performance of deep learning models [34]. The current dataset contained only 27.5 min of useful data, and such audio length became smaller when the dataset was split based on ingestive behaviors and forage characteristics. The relatively small ingestive sound dataset is typical of studies with dairy cows (i.e., 13 min in the study of Chelotti et al. [11]) because of their quick ingestive actions, challenging environment for data collection, laborious manual labeling, etc. A large ingestive sound dataset is recommended to be built in the future to improve model performance.

Forage characteristics also influenced the automatic classification of ingestive sounds. The best and poorest performance difference for classifying the ingestive behaviors was 0.4–0.5 among different forage characteristics. Chelotti et al. [11] reported a 0.11–0.41 performance difference and Milone et al. [14] demonstrated a 0.11–0.33 performance difference for classifying the ingestive behaviors under similar forage characteristics used in this study. Apart from uneven class balance and small datasets, similar acoustic features between the alfalfa and tall fescue or the two forage heights could decrease the classification performance. This may indicate that current techniques may not be sufficient or generalizable to differentiate the ingestive behaviors for various forage characteristics. When sound classification for specific forage is needed, the model may need re-development with custom datasets for robust classification. 

## 5. Conclusions

Classification of the three ingestive behaviors (bites, chews, and chew-bites) of dairy cows using deep learning models was conducted in this study. The results showed that forage species (alfalfa vs. tall fescue) and heights (tall and short) significantly influenced the amplitude and duration of the ingestive sounds of dairy cows. The LSTM using a filtered dataset with balanced duration and imbalanced audio files had better performance than its counterparts. Currently, it is difficult to differentiate the bites, chews, and chew-bites between alfalfa and tall fescue under two different heights. In addition to training the LSTM with more temporal data, sophisticated feature extraction techniques will be considered in a future study.

## Figures and Tables

**Table 1:** Number and duration of audio files used for model evaluation and experiments.

Forage Species	Forage Height	Number of Audio Files			Duration of Audio Files Used (s)		
		Bites	Chews	Chew-Bites	Bites	Chews	Chew-Bites
Alfalfa	Short	179	260	123	72.78	74.24	71.99
	Tall	148	416	322	175.20	184.56	182.90
Tall fescue	Short	94	454	217	143.87	144.79	141.59
	Tall	100	487	238	155.19	149.78	150.48
Total		521	1617	900	547.04	553.37	546.96

**Table 2:** Amplitude and duration of the bite, chew, and chew-bite sound under various forage conditions.

Factors	Bite		Chew		Chew-Bite	
	Amplitude	Duration (s)	Amplitude	Duration (s)	Amplitude	Duration (s)
Forage species						
Alfalfa	0.355b	0.176b	0.105	0.110b	0.389b	0.262b
Tall fescue	0.454a	0.208a	0.105	0.132a	0.520a	0.301a
SEM	0.012	0.004	0.002	0.003	0.008	0.004
Forage height						
Tall	0.403	0.206a	0.117a	0.138a	0.464	0.297a
Short	0.406	0.178b	0.093b	0.105b	0.446	0.266b
SEM	0.012	0.005	0.002	0.003	0.009	0.004
Interaction						
Alfalfa-Tall	0.387b	0.200a	0.127a	0.148a	0.435c	0.294a
Alfalfa-Short	0.323c	0.152b	0.084c	0.073c	0.343d	0.230b
Tall fescue-Tall	0.420b	0.212a	0.107b	0.128b	0.492b	0.301a
Tall fescue-Short	0.488a	0.205a	0.102b	0.137ab	0.549a	0.301a
SEM	0.017	0.006	0.003	0.005	0.012	0.005
*p*-Value						
Forage species	<0.01	<0.01	0.79	<0.01	<0.01	<0.01
Forage height	0.89	<0.01	<0.01	<0.01	0.16	<0.01
Forage species × Forage height	<0.01	<0.01	<0.01	<0.01	<0.01	<0.01

a,b,c,d Values within the same treatment groups with different letters aside indicate significant difference exists among the treatment means (*p* ≤ 0.05) according to Fischer’s LSD test. SEM is pooled standard error of the least square means. Amplitude is the normalized amplitude (unitless).

**Table 3:** *Precision*, *recall*, and *F*1 *score* of the three deep learning models for classifying the ingestive behaviors in various datasets.

Model	Behavior	Original-Imbalanced			Original-Balanced			Filtered-Imbalanced			Filtered-Balanced		
		*Precision*	*Recall*	*F*1 *Score*	*Precision*	*Recall*	*F*1 *Score*	*Precision*	*Recall*	*F*1 *Score*	*Precision*	*Recall*	*F*1 *Score*
Conv1D	Bite	0.782	0.819	0.800	0.827	0.819	0.823	0.783	0.790	0.786	0.837	0.781	0.808
	Chew	0.893	0.901	0.897	0.753	0.638	0.691	0.865	0.932	0.897	0.667	0.857	0.750
	Chew-bite	0.779	0.744	0.761	0.615	0.714	0.661	0.818	0.700	0.754	0.683	0.533	0.599
	Overall	0.840	0.841	0.840	0.731	0.724	0.725	0.837	0.839	0.838	0.729	0.724	0.726
Conv2D	Bite	0.810	0.810	0.810	0.851	0.819	0.835	0.728	0.867	0.791	0.844	0.876	0.860
	Chew	0.900	0.920	0.910	0.823	0.752	0.786	0.941	0.886	0.913	0.820	0.867	0.843
	Chew-bite	0.821	0.789	0.805	0.712	0.800	0.753	0.821	0.817	0.819	0.821	0.743	0.780
	Overall	0.861	0.862	0.861	0.795	0.790	0.792	0.869	0.862	0.865	0.828	0.829	0.828
LSTM	Bite	0.829	0.829	0.829	0.855	0.895	0.874	0.820	0.867	0.843	0.881	0.848	0.864
	Chew	0.935	0.929	0.932	0.767	0.848	0.805	0.935	0.895	0.915	0.864	0.905	0.884
	Chew-bite	0.841	0.850	0.845	0.831	0.705	0.763	0.824	0.861	0.842	0.837	0.829	0.833
	Overall	0.889	0.888	0.888	0.818	0.816	0.817	0.883	0.880	0.881	0.860	0.860	0.860

Notes: Conv1D is one-dimensional convolutional neural network; Conv2D is two-dimensional convolutional neural network; and LSTM represents long short-term memory network. “Original” indicates the original dataset without any filtering; “Filtered” indicates the original dataset was filtered with the bandstop filter to remove background beeping sounds; “imbalanced” indicates the dataset with unequal audio file sizes for the three ingestive behaviors; and “balanced” indicates the dataset with equal audio file sizes for the three ingestive behaviors.

**Table 4:** *Precision*, *recall*, and *F*1 *score* for classifying the ingestive behaviors under various forage conditions.

Behavior	Forage Species	*Precision*	*Recall*	*F*1 *Score*	Behavior	Forage Height	*Precision*	*Recall*	*F*1 *Score*
Bite	Alfalfa	0.742	0.697	0.719	Bite	Short	0.590	0.418	0.489
	Tall fescue	0.630	0.436	0.515		Tall	0.500	0.480	0.490
Chew	Alfalfa	0.720	0.753	0.736	Chew	Short	0.594	0.603	0.599
	Tall fescue	0.784	0.835	0.809		Tall	0.771	0.723	0.746
Chew-bite	Alfalfa	0.838	0.801	0.819	Chew-bite	Short	0.723	0.804	0.761
	Tall fescue	0.851	0.905	0.877		Tall	0.746	0.779	0.762
Overall		0.793	0.797	0.795	Overall		0.694	0.698	0.696

Notes: The model performance was investigated with the long short-term memory model and the filtered and imbalanced dataset.

**Table 5:** Performance comparison for classifying bites, chews, and chew-bites of dairy cows among different studies.

Positive Performance			Reference
Bites	Chews	Chew-Bites	
0.728–0.895	0.638–0.941	0.533–0.861	Current study
0.620–0.900	0.880–0.990	0.430–0.940	[11]
0.760–0.900	0.880–0.990	0.610–0.940	[14]
--	0.670–0.990	--	[23]

Notes: The positive performance includes accuracy, *precision*, *recall*, and *F*1 *score*. “--" indicates missing information.

## Data Availability

The acoustic dataset of dairy cows is available at https://github.com/sinc-lab/dataset-jaw-movements (accessed on 15 July 2021).
